# Coronary artery aneurysm presenting as ST-elevation myocardial infarction in a man with coronavirus disease 2019: a case report

**DOI:** 10.1186/s13256-022-03693-0

**Published:** 2022-12-16

**Authors:** David O. Alao, Amani Alabdouli, Ala Jalabi

**Affiliations:** 1 Department of Internal Medicine, College of Medicine and Health Sciences, United Arab Emirates University, Al Ain, United Arab Emirates; 2 Department of Emergency Medicine, Tawam Hospital, Al Ain, United Arab Emirates

**Keywords:** Coronary artery aneurysm, Acute STEMI, COVID-19, Healthcare system restructuring

## Abstract

**Background:**

Patients with underlying cardiovascular risk factors have worse clinical outcomes when they have coronavirus disease. In addition, a reduced workload of cardiovascular emergencies has been reported during the coronavirus pandemic due to patients’ reluctance to attend hospitals for fear of contracting the disease. Regional health service reorganization, separating hospitals into coronavirus and non-coronavirus can mitigate this effect. However, the effectiveness of this approach on outcomes and patient satisfaction is unknown.

**Case presentation:**

A 35-year-old Pakistani man with acute ST myocardial infarction was found to have thrombosis of the right coronary artery aneurysm and concomitant coronavirus disease. He had percutaneous coronary angiography and thrombus removal, and was transferred to a coronavirus hospital for the management of the infection. Due to the large size of the aneurysm, he was considered for surgical intervention. Following discharge from the coronavirus hospital and a period of stay at the isolation center, he failed to keep his cardiology follow-up appointment.

**Conclusion:**

This case illustrates an unusual cause of myocardial infarction in a patient with coronavirus infection whose care may have been adversely affected by the healthcare system restructuring.

## Background

The recent coronavirus disease 2019 (COVID-19) pandemic has had a major impact on healthcare provision and resource utilization. COVID-19 patients with underlying cardiovascular risk factors have worse clinical outcomes [1]. In addition, reduced cardiovascular emergency workload due to patients’ reluctance to attend hospitals for fear of contracting COVID-19 has been reported [2]. One way to mitigate this is regional health service reorganization, separating hospitals into COVID-19 and non-COVID-19. The effectiveness of this approach on clinical outcomes and patient satisfaction is unknown.

Our city has two major hospitals, and both receive acute cardiovascular emergencies. Following the outbreak of the COVID-19 pandemic, the hospitals were redesignated as COVID and non-COVID. All patients with COVID-19 disease are managed at the COVID hospital. Patients who present at the non-COVID hospital with a cardiovascular emergency such as ST myocardial infarction underwent percutaneous coronary intervention before transfer to the COVID hospital if they had a positive polymerase chain reaction (PCR) test.

We present a case report of a man with acute ST-elevation myocardial infarction resulting from coronary artery aneurysms and concomitant COVID-19, whose care may have been compromised by the regional healthcare reorganization as a result of the COVID-19 pandemic.

## Case presentation

A 35-year-old Pakistani man presented to our emergency department with a 1 h history of central chest pain radiating to the left arm with associated nausea and diaphoresis.

He was an ex-smoker of 10 pack/years, and he suffered from hyperlipidemia, for which he had been on lifestyle modifications and statin for the last 2 years. He had a positive family history of coronary artery disease. He denied any history of acute illness or hospital admission in his childhood.

**Fig. 1:**
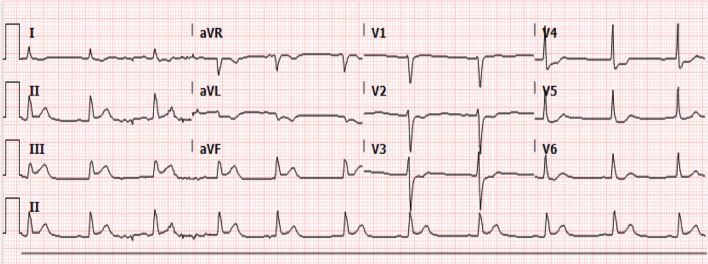
Twelve-lead electrocardiogram (ECG) showing acute inferior ST elevation

On examination, the patient was sweating and in pain, with a pain score of 8/10. He was conscious and alert with no neurological deficit. His initial vital signs were as follows: heart 63 beats per minute, regular; blood pressure, 126/81 mmHg, and respiratory rate of 20 per minute. He was afebrile, and had normal breath sounds and normal heart sounds with no murmur. His abdominal examination was normal. His 12-lead electrocardiogram showed ST-segment elevation in the inferior leads, with reciprocal changes in the anterior charges (Fig. 1). A diagnosis of an acute ST-elevation myocardial infarction was made, and he was transferred for percutaneous coronary intervention. He received 5000 units of unfractionated heparin, 300 mg of aspirin, 80 mg of atorvastatin, 180 mg of ticagrelor, and 5 mg of intravenous morphine.

Laboratory findings were remarkable for initial troponin T 9.6 ng/L (< 14 ng/L), low-density lipoprotein 6.56 mmol/L (< 3.36 mmol/L), triglyceride 1.73 mmol/L (0.45–1.69 mmol/L), cholesterol/high-density lipoprotein 9.96 (3.35).

A portable chest radiograph was normal.

**Fig. 2:**
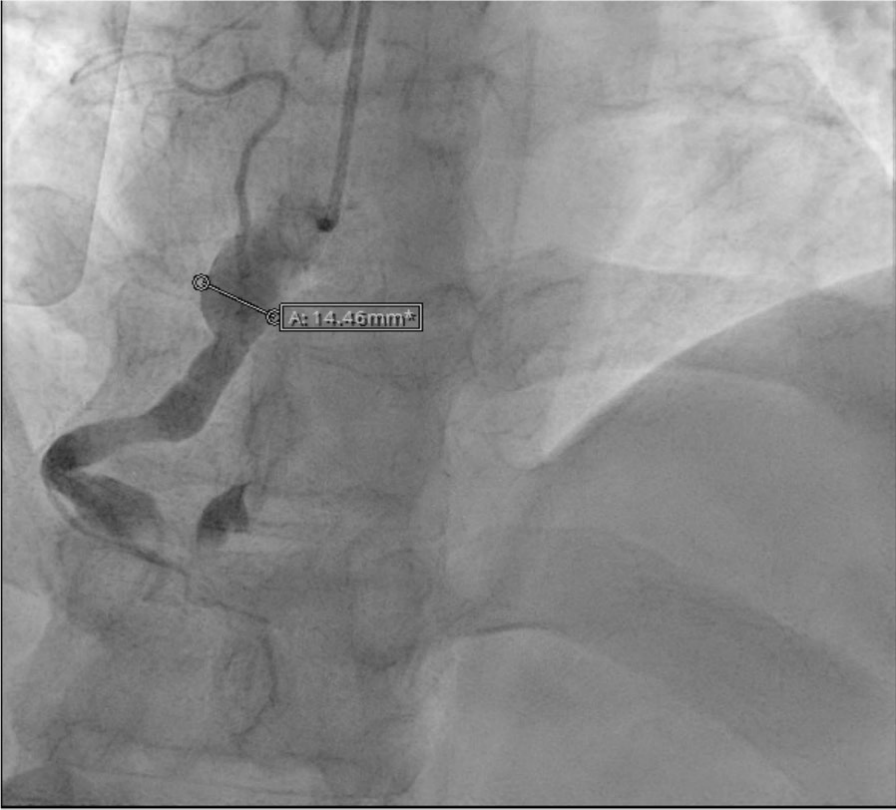
Right coronary artery aneurysm with occlusive thrombus

**Fig. 3:**
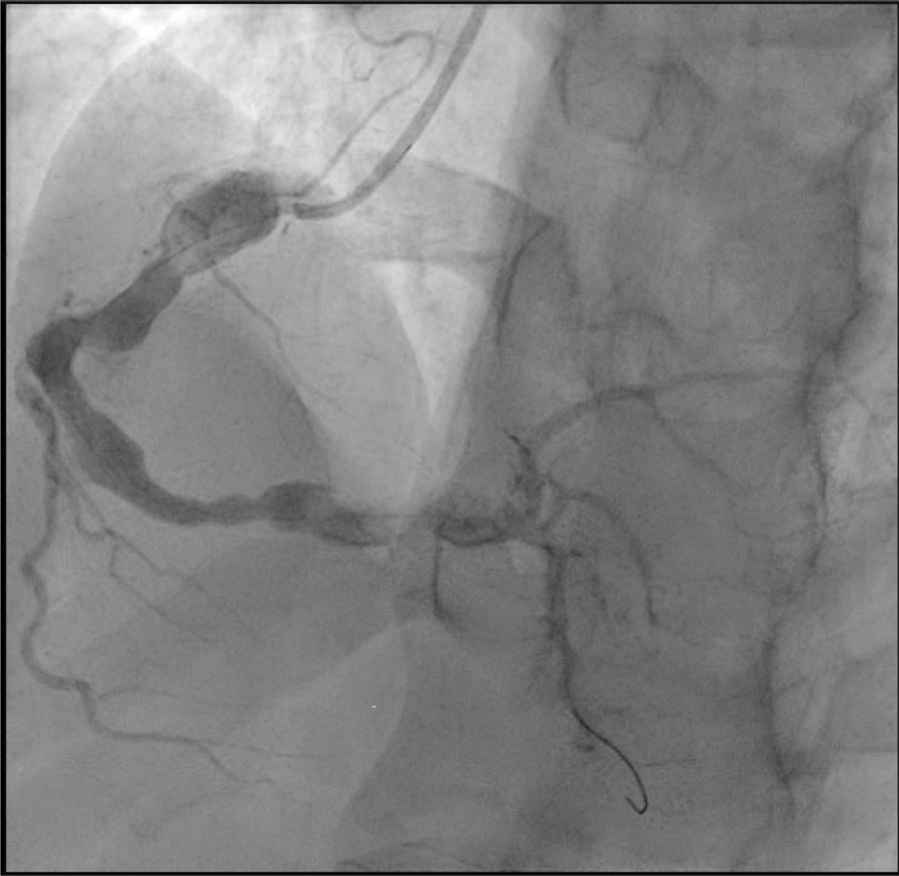
Right coronary artery aneurysm with TIMI grade 2 flow after thrombus retrieval

His angiogram showed a medium-size aneurysm in the circumflex artery, and a large aneurysm (14.46 mm) in the proximal right coronary, followed by multiple sequential aneurysms and 100% clot occlusion (Fig. 2). His left main and anterior descending arteries were normal. The clot was removed with an aspiration catheter. Thrombolysis in myocardial infarction (TIMI) grade 2 flow was established (Fig. 3). The ostium was dilated but not stented. He was placed on statin, ezetimibe, angiotensin-converting enzyme inhibitor (ACE-I), beta-blocker, and dual antiplatelet therapy.

Six hours later, and while recovering in the high dependency unit, his polymerase chain reaction (PCR) test for COVID-19 came back positive. The patient was informed of the findings of his PCR and the result of his positive COVID-19 PCR test. He was also made aware of the medical and surgical options for the management of his coronary artery aneurysms and that these would be discussed with him after he had recovered from COVID-19. The hospital policy for managing COVID-19 was explained to him, and he was subsequently transferred to the regional COVID-19 hospital. This was in accordance with our healthcare service reorganization plan to manage all COVID-19 patients at dedicated facilities to protect the non-COVID hospitals. He remained asymptomatic at the COVID hospital and was later discharged to an isolation facility. He did not keep his cardiology clinic follow-up appointment at our institution on two separate occasions. Following a telephone inquiry, he claimed to be stressed due to his experience at the COVID hospital and was unwilling to return for a follow-up. He presented again to the emergency department of our hospital 2 months after with symptoms suggestive of an acute stress reaction secondary to COVID-19 infection and hospital experience. He was managed for stress and was discharged home. The patient has provided a written consent for this publication.

## Discussion

Our patient had an unusual combination of acute STEMI resulting from thrombotic occlusion of the right coronary artery aneurysm and concomitant COVID-19. This combination led to competing clinical priorities and a potentially poor outcome for the patient.

The incidence of acute myocardial infarction (MI) in patients with COVID-19 is 4.2%, and there is evidence that COVID-19 worsen outcomes in patients with cardiovascular diseases [1]. In addition, COVID-19 can precipitate cardiac injuries such as nonspecific myocardial injury, coronary spasm, non-ischemic cardiomyopathy, and myocardial infarction [3].

Coronary artery aneurysm (CAA) occurs in approximately 4.5% of coronary artery angiographies. It is associated with arteriosclerosis in older patients, but in children, it is often the result of Kawasaki disease, where up to 20% of incidences have been reported in untreated cases [4]. Other causative factors are Takayasu arteritis, coronary artery stent complications, and genetics [5]. Coronary artery aneurysm is often an incidental finding but can occasionally present with complications such as angina, rupture, and STEMI. The reduced blood flow in the aneurysm predisposes to clot formation, leading to partial or complete occlusion of flow distal to the aneurysm. The cause of the patient’s CAA is uncertain. He denied any history of hospital admissions as a child that could suggest Kawasaki disease.

Furthermore, his coronary angiography did not show arteriosclerosis. The patient developed an acute STEMI due to thrombotic occlusion of his right coronary artery. Increased venous and arterial thrombotic complications have been reported in patients with COVID-19 disease [6]. In addition, COVID-19 patients with acute STEMI have an increased thrombus load and higher rates of stent occlusion [7, 8]. It is uncertain how COVID-19 disease contributed to the development of thrombosis in our patient.

Management of CAA is on an individual basis due to a lack of consensus on the best treatment strategy. Small aneurysms (< 10 mm) are treated medically with dual antiplatelet and anticoagulation, while large aneurysms are managed with covered stents [5]. Giant aneurysms require surgical management [9]. Our patient was initially treated medically to expedite his transfer to the COVID-19 hospital. Based on the size of his aneurysm, he was a potential candidate for other interventions, such as covered stent and surgery. However, these options were not fully explored with him as he was lost to cardiology follow-up.

This case illustrates a particular challenge posed by the COVID-19 pandemic. Many emergency services have experienced reduced attendance during the pandemic [10]. A common reason for these is the fear of contracting COVID-19 in the hospital. A similar scenario may play out in high-risk patients who require follow-up. Moreira *et al.* recently reported a 13% reduction in the follow-up of patients with high-risk cardiovascular conditions [11]. The impact of this observation on patient’ outcomes is unknown, and further studies are required. Our region adopted a system similar to that used in China and Italy, which centralized the care of COVID-19 patients in COVID hospitals, while keeping other hospitals free from the disease [12, 13]. A potential downside is that care of non-COVID-19 diseases may become compromised when such patients are managed in a COVID-19 facility.

## Conclusions

Acute myocardial infarction can be precipitated directly and indirectly by COVID-19. Those with underlying cardiovascular conditions are at high risk. Additional efforts must be made to mitigate the reasons for the nonattendance of high-risk patients. Health care reorganization in the face of the COVID-19 pandemic may negatively impact the clinical outcome and patient satisfaction.

## Data Availability

Not applicable.

## Abbreviations

CAA: Coronary artery aneurysm
COVID-19: Coronavirus disease 2019
MI: Myocardial infarction
PCR: Polymerase chain reaction
TIMI: Thrombolysis in myocardial infarction
STEMI: ST-elevation myocardial infarction
